# Swedish Intensive Care Nurses’ Knowledge, Attitudes, and Views on Donation After Circulatory Death Before a National Implementation *

**DOI:** 10.1177/23779608241274208

**Published:** 2024-08-22

**Authors:** Emilie Gripewall, Lisbeth Fagerström, Christine Kumlien, Janet Mattsson, Linda Nyholm, Gunilla Björling

**Affiliations:** 1 Faculty of Education and Welfare Studies, Department of Health Sciences, Åbo Akademi University, Vaasa, Finland; 2 National Donation Center, 11247 National Board of Health and Welfare, Stockholm, Sweden; 3 Department of Care Science, Malmö University, Malmö, Sweden; 4 Department of Cardiothoracic and Vascular Surgery, Skåne University Hospital, Malmö, Sweden; 5 Faculty of Health and Social Sciences, 11310 University of South-Eastern Norway, Borre, Norway; 6 Department of Health Science, 4342 Kristianstad University, Kristianstad, Sweden; 7 Department of Neurobiology, Care Sciences and Society, 27106 Karolinska Institutet, Stockholm, Sweden; 8 Department of Caring and Ethics, University of Stavanger, Stavanger, Norway; 9 Department of Nursing, 4161 Jönköping University, School of Health and Welfare, Jönköping, Sweden; 10 Faculty of Nursing, 108094 Kilimanjaro Christian Medical University College, Moshi, Tanzania; 11 Department of Anaesthesia and Intensive Care, Södertälje Hospital, Södertälje, Sweden

**Keywords:** donation after circulatory death, organ donation, intensive care nurse, attitudes, knowledge

## Abstract

**Introduction:**

An increasing population and a shortage of identified potential organ donors are causing the waiting list for organ transplants to grow continuously. Donation after circulatory death (DCD) is a method aimed at meeting the demand for transplantable organs. However, it presents new challenges in nursing care, and there is a lack of studies investigating nurses’ attitudes and knowledge of DCD.

**Objective:**

The objective of this study was to determine and describe intensive care nurses’ (ICNs’) knowledge, attitudes, and views on DCD before a national implementation in Sweden.

**Method:**

This study utilized a cross-sectional mixed-method design. A convenience sampling method was employed, targeting ICNs working in four intensive care units in Sweden. A study-specific tool comprising fixed and free-text questions was developed. Fifty-one ICNs participated. Data were analyzed descriptively, and correlation analysis was performed using Spearman's correlation. Free-text answers were qualitatively assessed and analyzed. An integrated analysis was conducted to synthesize the quantitative and qualitative findings.

**Results:**

Fifty-three percent reported limited knowledge about DCD. Nurses with previous education on DCD had significantly higher knowledge (*r* = .380, *p* = .006), were more engaged with the public debate on organ donation (*r* = .423, *p* = .002), and considered the ethical aspects of DCD more thoroughly (*r* = .386, *p* = .022). The qualitative analysis identified four key categories: *The importance of the team, the need for ethical discussions, increased knowledge of DCD,* and *unanswered questions and unmet needs*. The integrated analysis underscored the need for targeted education, clear guidelines, and ongoing ethical discussions to prepare ICU nurses for DCD.

**Conclusion:**

Nurses educated in organ donor care had better knowledge and a more positive attitude toward DCD implementation. The study also highlights the importance of interprofessional teamwork in DCD implementation. The findings suggest that education on DCD could improve the identification and implementation of DCD donors, addressing the global shortage of transplantable organs.

## Introduction

An increasing population and a lack of identified potential organ donor patients make the waiting list for people needing a new organ constantly growing (Eurotransplant, 2022; Fleming & Thomson, 2018; Scandiatransplant, 2023). Organ donor care is, therefore, in need of reorganization in novel ways, and implementation of donation after circulatory death (DCD) is one way to achieve the demand for transplantable organs (National Board of Health and Welfare, 2023; Vanholder et al., 2021). Death and the regulation of declaring a patient dead are important parts of the organ donation process (Sade, 2011; the President's Council on Bioethics, 2008). Death is described in two different ways: death by neurological criteria, and circulatory death which require two different organ donation processes: donation by brain death (DBD) and DCD (Fleming & Thomson, 2018; Guzik-Makaruk et al., 2019; Manara et al., 2012; Spear et al., 2022). There are differences between DBD- and DCD donation (Leiden et al., 2016; Manara et al., 2012), but however, similar in both processes are a lack of blood flow to the brain, not compatible with a future life (the President's Council on Bioethics, 2008; The Swedish Board of Health and Welfare, The Swedish Code of Statutes [SOSFS], 2005:10). A DBD donor is a patient suffering from a catastrophic brain injury (Manara et al., 2012). Blood circulates the organs by the donor's blood circulation while the heart is still beating, but vital functions are maintained mechanically by a ventilator because the vital functions are completely absent due to brain damage (Sade, 2011; Spear et al., 2022). In the DBD process, death is declared by neurological criteria (Leiden et al., 2016, Spear et al., 2022) and the organ retrieval operation must start within 24 hours after the declaration of death is confirmed (SOSFS, 2005:10).

The DCD patient is a non-heart-beating donor patient (Sade, 2011). When intensive care treatment no longer seems meaningful for a future life, an end-of-life decision is taken by two physicians, and the palliative care phase begins. A circulatory arrest must then arise within three hours to qualify as a DCD donor (Kondori et al., 2021; Leiden et al., 2016; National Board of Health and Welfare, 2023). Thereafter, a 5-minute non-touch period follows before the patient can be declared dead. The organ retrieval, or pre-surgery perfusion of the abdominal organs (normothermic regional perfusion, NRP), must then start urgently, as the organs lack perfusion. The limitation of time is pivotal in DCD after the death of the patient (Hunt et al., 2022). The implementation of DCD places high ethical demands on nursing care as the patient is conscious when the DCD process starts. There is a lack of studies investigating nurses’ attitudes, and knowledge toward DCD. Therefore, it is important to determine and describe intensive care nurses’ (ICNs) knowledge, attitudes, and views on DCD.

## Literature Review

Today, DCD has started to become an established method for organ donation worldwide (Cooper et al., 2023; Eurotransplant, 2022; Fleming & Thomson 2018). In Sweden, national implementation is ongoing in hospitals and intensive care units (ICUs) after a pilot project year in 2019, and 68 DCDs were performed in 2023 in Sweden (National Board of Health and Welfare, 2023; Scandiatransplant, 2024; The Swedish National Council for Organs, Tissues, Cells and Blood, 2019). A new regulation from 2018 (The Swedish Code of Statutes [SFS], 2018, p. 307) regarding the Organ Registry at the National Board of Health and Welfare made it possible to search for patients’ statements regarding their wish to donate organs after death, also before the patient is declared dead (SFS, 2018, p. 307). Furthermore, a reform of the regulation that enables organ conservative treatment before the patient actuality is declared dead was established on 1 July 2022 (SFS, 2022, p. 582). The ethical discussions regarding the declaration of death and DCD donation is a new area to manage and discuss in the debate on organ donor care worldwide (Cooper, 2017, 2023; Guzik-Makaruk et al., 2019; Sade, 2011; Tantillo et al., 2019; Zeiler et al., 2008). The medical challenges and technical support in DCD donation, for example, NRP that restores oxygenated blood flow in the transplantable organs are today well covered in published articles (Alamouti-Fard et al., 2022; Foss et al., 2018; Hunt et al., 2022; Klosiewicz et al., 2017; Puslecki et al., 2017). However, there is a lack of studies describing caring for potential organ donor patients worldwide. Furthermore, studies on ICU nurses’ experiences of taking care of DCD patients and the multidimensional challenges that nurses face during the DCD process need to be promoted (Gripewall et al., 2022).

The care of an organ donor patient is described as a non-everyday task for ICU nurses. It is both stressful and complex and includes ethical challenges, struggles, and burdens for the nurses concerning their values and feelings and involves high demands when communicating with relatives (Cooper, 2023; Gripewall et al., 2022; Milross et al., 2022; Moghadam et al., 2018). However, to facilitate the implementation of DCD to a greater extent, knowledge about how ICU nurses perceive the DCD process would be beneficial. ICU nurses need to be prepared for a new complex caring situation both from an organizational point of view and from an ethical perspective. DCD will be an additional way to manage the donor process in Sweden, with new challenges for the ICU nurses to handle during the complex multidimensional donor process (National Board of Health and Welfare, 2023; Rosen et al., 2018). Therefore, the main objective of this study was to determine and describe Swedish ICU nurses’ knowledge, attitudes, and views on DCD before a national implementation of the DCD process in Sweden.

## Methods

### Design

This study has a cross-sectional mixed-method design, integrating both quantitative and qualitative approaches to comprehensively understand Swedish ICU nurses’ knowledge, attitudes, and views on donation after circulatory death (DCD) before its national implementation in Sweden. The design was chosen to capture the breadth and depth of the topic (Creswell & Plano Clark, 2018). The cross-sectional design examines knowledge and attitudes at a specific point in time, which was important before the national implementation of the DCD process (Polit & Beck, 2022).

### Research Questions

- What are ICU nurses’ knowledge and attitudes toward the DCD process before implementation?
- What are the associations between nurses’ background variables, knowledge, and attitudes?
- What are nurses’ views on DCD?

### Setting

The ICUs in the present study were chosen by the Regional Center of Organ Donation (RDC) in 2019, Sweden. The chosen ICUs (*n* = 4) had a commanded plan to implement DCD following the national DCD project (The Swedish National Council for Organs, Tissues, Cells and Blood, 2019). All ICUs were located in district hospitals in larger cities in Sweden.

### Eligible Criteria

#### Inclusion Criteria

The inclusion criteria were ICU nurses who worked in an ICU designated for a commanded plan to implement DCD following the national DCD project (The Swedish National Council for Organs, Tissues, Cells and Blood, 2019).

#### Exclusion Criteria

Other healthcare professionals, such as physicians or assistant nurses, were excluded.

### Data Collection

#### Sampling

**Figure 1.**
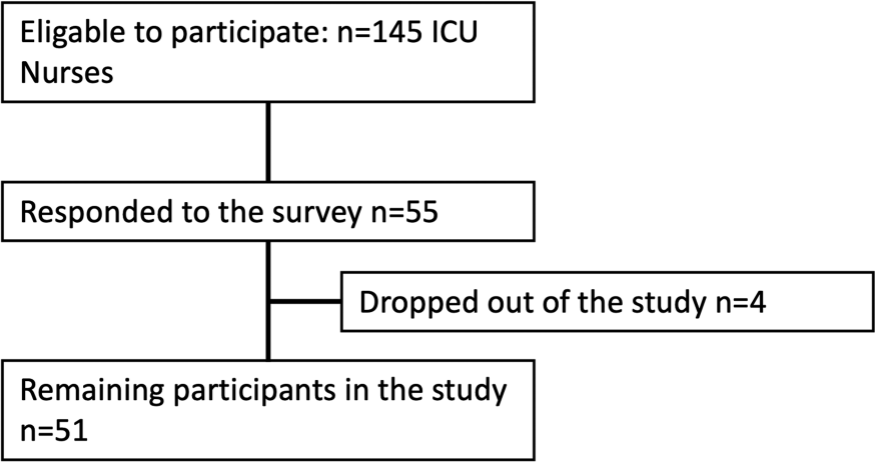
Flowchart of the sampling of participants.

An invitation letter and information about the study were sent to the head nurse of four ICUs, chosen after discussion with the Swedish National Council for Organs, Tissues, Cells, and Blood. However, three of those units chose to participate in the study. After acceptance, a link to the web survey was sent out in November 2019 to the head nurses of the ICUs, which thereafter, sent the link together with an information letter to all ICU nurses at each participating ICU unit (*n *= 145 nurses). Thereby, the anonymity of the participants was maintained. Of the 145 ICU nurses, 55 chose to answer and four (4) did not fulfill the questionnaire rendering 51 participants in the study, see Figure 1. The response time was set to 2 weeks and one reminder was sent out after one week, to increase the response rate (Polit & Beck, 2022). Participation was voluntary, all the collected data was anonymous and could not be traced to a specific person. The participants gave their informed consent before participating by continuing to answer the survey.

#### Study-Specific Tool

A validated tool about nurses’ knowledge and attitudes toward DCD was not found at the time of the study start. Therefore, a study-specific tool was developed and used in the study. The content was based on (a) discussions from meetings regarding DCD within the medical context, (b) the results from the national DCD pilot project phase I (The Swedish National Council for Organs, Tissues, Cells and Blood, 2019), and (c) previous publications (Manara et al., 2012; Moghadam et al., 2018). All researchers who developed the tool held a PhD degree except one, who had an MSc and the development of the questionnaire was made in alignment with Polit & Beck (2022).

The tool was divided into 4 (four) parts and consisted of 26 items. The first part consisted of five (5) questions which were demographic, that is, age, gender, work experience as an ICU nurse, experience of caring for a donor patient, as well as post-graduate organ donor education. The second and third parts consisted of questions about knowledge and attitudes toward DCD, that is, knowledge about the DCD process, and the implementation, as well as their view on the differences between DBD and DCD in different donor process scenarios. The last part consisted of voluntary open-ended questions. When the interest of the subject is highlighted, the validity of the results has higher trustworthiness (Bernard, 2013; Boynton & Greenhalgh, 2004; Polit & Beck, 2022). In conclusion, there were 5 (five) demographic questions, 17 questions for the second and third parts, and the fourth part included four (4) questions with a free-text possibility. All questions were mandatory to answer except the open-ended questions. Regarding the types of questions, three (3) questions were of a Likert's scale type, 14 questions had fixed answers, four (4) questions were open-ended, and five (5) questions had multiple choices including two (2) questions with free-text options, see Table 1.

**Table 1.** Types of Questions (*n* = 26).

Type of question	No. of items
Likert's scale	3
Multiple options	2
Multiple options with a free-text option	3
Fixed answers Yes/No	3
Fixed answers Yes/No with a free-text option	11
Free-text only	4

##### Pre-Test and Face Validity

A pre-test of the study-specific tool, prior to the initiation of the study, was carried out on six ICU nurses from three different ICUs who did not participate in the study. The pre-testing was made to secure that the tool was user friendly and understood, but also to confirm the correspondence between the questions and the objectives of the study in accordance with Polit and Beck (2022). After the pre-test, the questionnaire was slightly modified. Only minor language changes in two questions regarding attitudes toward DCD were made to the tool to make the context easier to understand, as suggested by Boynton (2004). Face validity, was thereafter, done by six other nurses after the minor alterations of the tool.

### Ethical Considerations

The study was performed following the ethical principles established by the Declaration of Helsinki (World Medical Association, 2013) and followed the Swedish law for ethical vetting (2003:460). The protocol was ethically approved. No personal information was collected, and all data were collected anonymously. Permission to collect the data was received from the head nurse of each department. The participants were informed that their participation in the study was voluntary and provided informed consent. The data cannot be traced to a specific person, and the results are presented on group-level and the excerpts cannot be traced to a specific person as they were collected anonymously.

### Data Analysis

#### Statistical Analysis

A quantitative, descriptive analysis was performed. The data was univariately analyzed. The variables were both nominal and ordinal and the results were reported in numbers and percentages. Background variables such as age, working experience and education were reported in terms of the means and range. To explore correlations between the background variables, knowledge, and attitudes, correlation analyses were performed with Spearman correlation rho. A level of *p* < .05 was considered significant*.* As most questions were of ordinal data the Spearman Correlation test was the most applicable correlation test to use (Moxham, 2012; Polit & Beck, 2022). To test the reliability of the study-specific tool Cronbach's Alpha was calculated for the three items using Likert's Scale. *Statistical Package for the Social Sciences version 27.0 (SPSS)* was used for the analyses.

#### Qualitative Analysis

The open free-text answers and questions with the possibility to add a comment were qualitatively assessed and analyzed inspired by Graneheim and Lundman's (2004) content analysis. Firstly, the free-text answers were sorted manually. The data was, thereafter, analyzed by breaking down the text into smaller units, coding, and naming the units according to the content in the text. The meaning units were then categorized into four main categories and agreement was reached. The first author made the analysis which was verified by two co-authors. The categories were then discussed in the large research group.

#### Integrative Analysis

The integration of quantitative and qualitative results was carried out through a convergent mixed-methods design, where both data sets were collected simultaneously but analyzed separately before being integrated during the interpretation phase (Creswell & Plano Clark, 2018; Polit & Beck, 2022). Firstly, the findings from both the quantitative analysis and the qualitative analysis were compared and contrasted to get an understanding of the results. The level of integration (Creswell & Plano Clark, 2018) in the analysis went from independent to integrated and the analysis aligned with the research questions. The integration analysis was carried out by the first author and discussed within the research group.

#### Data Quality Measures

To ensure the data collected in the present study the following data quality validity was done. We used face validity of the study-specific research tool by piloting the tool in six nurses to ensure its comprehensibility. Regarding authenticity, we developed the tool based on present research in the field as there was not a validated tool that the researchers were aware of. To determine the internal consistency of the tool, Cronbach's Alpha was calculated for the three Likert's Scale questions. The integrity was maintained as the data collection was done anonymously and it was not possible to identify the participants of the study. Criticality was obtained as the study-specific tool was based on current research in the field and distributed in ICU settings familiar with organ donation. To ensure the credibility of the statistical findings we used appropriate statistical tests and to make a correct interpretation of the results (Moxham, 2012; Polit & Beck, 2022). Regarding the qualitative analysis of the open-ended questions, trustworthiness: credibility, dependability, transferability, and confirmability were applied to strengthen the results of the study, which are recommended by Lincoln and Guba (1985). Authenticity (Guba & Lincoln, 1985) was also considered as the data collection was carried out in ICUs where organ donor patients are usually cared for.

## Results

**Figure 2.**
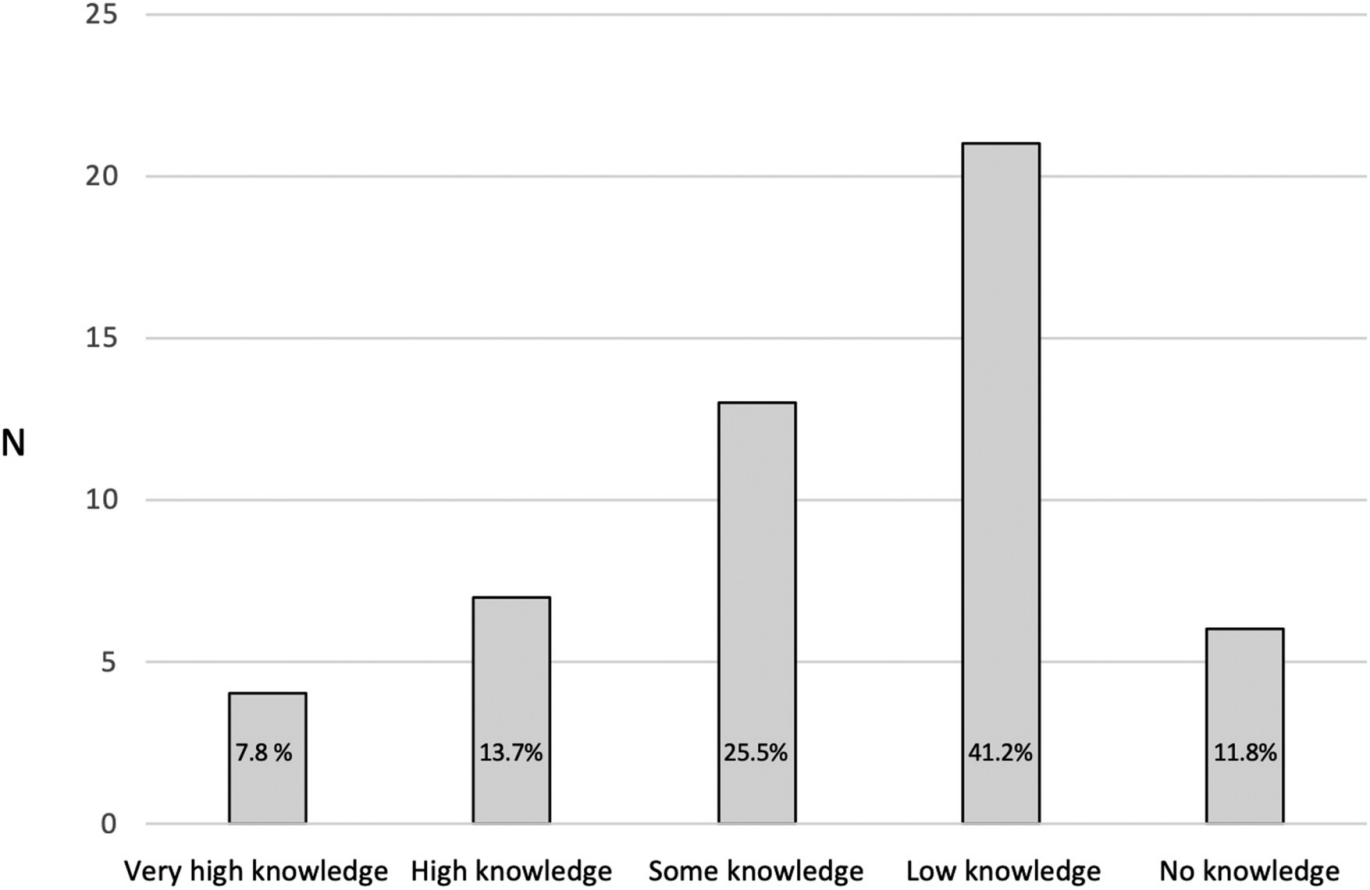
Participants’ knowledge about DCD (*n *= 51; “What level of knowledge do you have of Donation after Circulatory Death (DCD)?”).

The questionnaire was sent out to *n* = 145 ICU nurses at three ICUs in Sweden*.* The response rate was 38% (*n = 55*). However, four (*n *= 4) of the participants decided to start but did not complete the survey and were, therefore, excluded from the analysis, rendering *n* = 51 (35%) participants in the study, see Figure 2. The reliability test using Cronbach's Alpha for the three items that used a Likert's Scale (*Q20 How important do you think it is to have physician involvement in the care of a DCD patient?, Q9 What level of knowledge do you have of DCD?,* and *Q10 To what extent have you followed the public debate on DCD?)* showed an agreeable reliability Cronbach's *α* = 0.517.

### Sample Characteristics

The characteristics of the participants are displayed in Table 2. As stated in Table 2, all participants *n *= 51 were registered ICU nurses, where *n *= 44 (86%) were women and *n *= 7 (14%) men. The mean age was 46.7 years, the mean years working as a nurse was 21.4 years, and the mean number of years working in an ICU was 15 years. Furthermore, almost all participants *n *= 49 (96%) reported experiences caring for a potential organ donor patient. The result shows that *n *= 28 (55%) had completed organ donor education by Organisation for Organ Donation (OFO). The European Donor Hospital Education Programme (EDHEP) was the second most frequent organ donor-specific education among the participants *n = *14 and *n *= 7 of the participants had attended other types of organ donor education.

**Table 2.** Demographic Data of the Participants (*n *= 51).

Variable	*N* (%)	Range	Mean	SD
Age	51	31–66	46.7	±10.0
Sex				
Male	7 (13.7)			
Female	44 (86.3)			
Experience				_
No. of years as a registered nurse	51	4–41	21.4	±10.0
No. of years in ICU	51	2–35	15.0	+9.3
Cared for an organ donor patient				
Yes	49 (96.1)			
No	2 (4)			
Has organ donor education				
OFO^a^	28 (54.9)			
EDHEP^b^	14 (27.4)			
Other organ donor education	7 (13.7)			

^a^ Organisation for Organ donation (OFO).

^b^ The European Donor Hospital Education Programme (EDHEP).

### Knowledge of Donation After Circulatory Death

Regarding the nurses’ knowledge of DCD, more than half of the ICU nurses *n *= 27 (53%) had limited knowledge of DCD, with *n *= 21 (41.2%) reporting having low knowledge about DCD and *n *= 6 (11.8%) reporting no knowledge about DCD. However, *n* = 4 (7.8%) of the respondents reported having very high knowledge and *n* = 7 (13.7%) reported having high knowledge about DCD, see Figure 2.

The number of nurses who had unanswered questions about the DCD process was *n* = 26 (50.9%), while *n *= 25 (49.1%) reported the opposite. Knowledge about the new regulations on organ donor consent (SFS, 2018, p. 307) was 59% (*n *= 30), see Table 3.

**Table 3.** Participants’ Knowledge and Attitudes of the DCD—Process (*n* = 51).

*Variable*	*Yes* *n (%)*	*No* *n (%)*
*Knowledge about DCD*		
*Having unanswered questions about DCD*	26 (50.9)	25 (49.1)
*Having knowledge about the new organ donation registry regulations (**SFS, 2018*, p. *307)*	30 (58.8)	21 (41.2)
*Having DCD discussion at the unit*	32 (62.7)	19 (37.3)
*Attitudes toward DCD*		
*Possibility of DCD implementation at your unit*	51 (100)	0
*Possibility of more identified organ donor patients if implement DCD*	51 (100)	0
*The possibility that DCD implementation can be a reality*	51 (100)	0

### Attitudes to Donation After Circulatory Death

**Figure 3.**
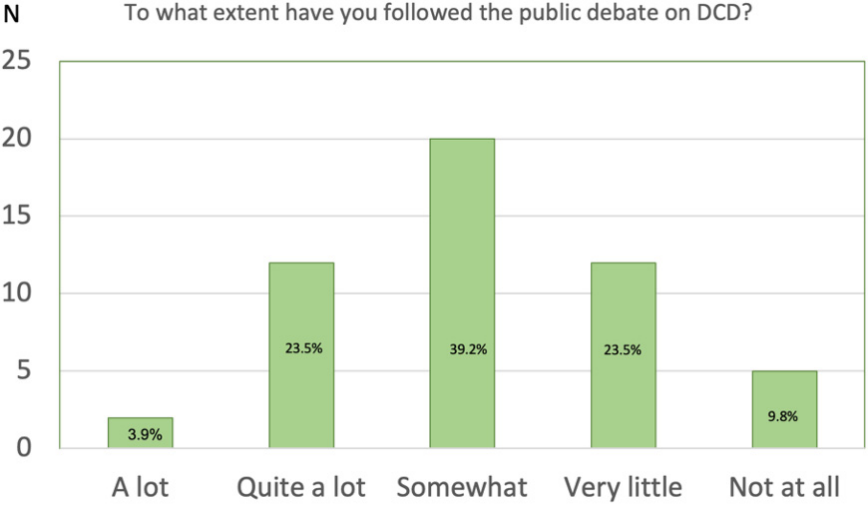
The participants’ attitudes to follow the public debate on DCD.

Almost 40% (*n* = 20) of the nurses in the present study followed the public debate on DCD somewhat; however, more than a third of the nurses *n *= 17 stated that their interest in the public debate on DCD was very low (*n *= 12) or non-existent (*n *= 5), see Figure 3.

The nurses highlighted anticipated differences in four areas regarding the process of caring for DCD patients compared to DBD patients (caring team, relatives, caring, and identification). In three areas: the caring team, caring for relatives, and caring for the patient, most nurses believed in no or small differences between the different ways of caring for DCD patients compared to DBD patients. Concerning the process of the identification of an organ donor patient, 71% thought there would be differences between DBD and DCD donation identification, see Table 3. Regarding the question about the importance of the physician's commitment to the care of the DCD patient, *n *= 48 (94.1%) of the respondents highlighted this area as very important and *n *= 3 (3.9%) considered it as important.

The nurses highlighted thoughts about the practical process of DCD, see Table 4. Areas that were discussed in their units included, that is, a wish for implementation (*n* = 18), but also practical questions about the implementation (*n* = 17), ethical discussions (*n *= 13) and personal thoughts about DCD (*n *= 14) as the focus of discussions at the ICUs. Personal thoughts were represented from *interesting* (*n *= 38) to being unethical (*n *= 2).

**Table 4.** Nurses’ Views on Anticipated Differences Regarding the DCD Process Compared to the DBD Process (*N *= 51; “Based on Your Knowledge, Do You Think There Will be Any Differences in These Areas Between DBD and DCD?”).

Areas	Yes *n* (%)	No *n* (%)
Composition of the care team	16 (31)	35 (69)
Caring for relatives	14 (27)	37 (73)
Caring for the patient	16 (31)	35 (69)
Identification of a donor	36 (71)	15 (29)

All nurses *n *= 51 (100%) stated the implementation of DCD as a possibility, and they agreed that DCD implementation could be a reality at the unit in the future, see Table 5.

**Table 5.** Areas of DCD That Have Been Discussed at the Respondents’ Units, Thoughts About the DCD Process and Importance of the Implantation Process (*n *= 51).

	***n***
**Q 14. Areas of DCD discussed at your unit** ^a^	
A wish for implementing DCD at the unit	18
Practical questions about an implementation of DCD at the unit	17
Personal thoughts about DCD	14
Ethical discussions about DCD	13
Specific patient cases	2
Other	8
*Total*	*72*
**Q 15. Thoughts about the DCD process** ^a^	
Interesting	38
Exciting	28
Instructive	22
Unethical	2
Other	8
*Total*	*104*
**Q 22. Importance of the implementation process** ^b^	
Interprofessional education	18
Exchange of knowledge from teams in the DCD project phase 1	15
Ethical discussions about DCD	10
Project information from the national DCD project	6
Other	2
*Total*	*51*

^a^ More than one answer is possible.

^b^ Only one answer is possible.

They also believed that the implementation of DCD would increase the number of possible organ donors from their units. One important factor regarding the implementation of DCD, mentioned by 29.4% (*n *= 15) of the nurses, was an exchange of experience from the already existing DCD team from the project phase, and 35% (*n *= 18) emphasized that interprofessional education was also important, see Table 5.

#### Associations Between Nurses’ Background Variables, Knowledge, and Attitudes

The correlation analysis, see Table 6, showed that both age (*r* = .296, *p* = .035) and the number of years as a nurse (*r* = .317, *p* = .023) correlated significantly with thoughts about caring for the DCD patient's relatives. Furthermore, the correlation analysis showed that respondents who had received education from OFO followed the public debate regarding organ donation (*r* = .423, *p* = .002), and they also had a higher level of knowledge about the DCD process (*r* = .380, *p* = .006). The results also show that those who had education from OFO significantly correlated positively with ethical interest regarding DCD (*r* = .386, *p* = .022) as well as a positive attitude toward implementation at their unit (*r* = .373, *p* = .027). Concerning those who had not received education about DCD, there was a negative correlation with unanswered questions about the DCD process (*r* = −.425, *p* = .002).

**Table 6.** Correlation Analysis.

Variable	Age	Number of years as a RN	No of years in ICU	Experience of caring for an organ donator	Education from Organisation For Organ donation (OFO)	Other organ nodnor education	What level of knowledge do you have of Donation after Circulatory Death (DCD)?
*Age*	1.000	,892^**^	,861^**^	−0.041	−0.147	−0.093	0.012
*Number of years as a RN*	,892^**^	1.000	,933^**^	−0.131	−0.152	−0.150	0.047
*No of years in ICU*	,861^**^	,933^**^	1.000	−0.206	−0.153	−0.157	0.041
*Experience of caring for an organ donator*	−0.041	−0.131	−0.206	1.000	0.020	0.169	0.227
*Education from Organisation For Organ donation (OFO)*	−0.147	−0.152	−0.153	0.020	1.000	0.198	,380^**^
*Other organ donor education*	−0.093	−0.150	−0.157	0.169	0.198	1.000	,346^*^
*What level of knowledge do you have of Donation after Circulatory Death (DCD)?*	0.012	0.047	0.041	0.227	,380^**^	,346^*^	1.000
*To what extent have you followed the public debate on DCD?*	−0.201	−0.176	−0.216	0.093	,423^**^	0.242	,512^**^
*Do you have unaswered questions refardning DCD?*	0.012	−0.045	−0.047	0.004	−0.258	−0.216	−,425^**^
*Are you aware of the change in legislation in Sweden that enables the identification of DCD donors?*	−0.211	−0.099	−0.121	0.036	,283^*^	0.133	,338^*^
*Is DCD something discussed in your department?*	−0.006	0.160	0.170	−0.156	,280^*^	,315^*^	,298^*^
*Ethical issues.*	0.000	0.164	0.094	−0.066	,386^*^	−0.037	,366^*^
*Possibility to implement DCD.*	0.238	0.139	0.094	0.007	,373^*^	0.144	,389^*^
*Practical issues regarding implementation.*	−0.218	−0.196	−0.201	0.239	0.210	,428^*^	,339^*^
*Personal opinion about DCD.*	0.026	0.159	0.127	0.201	0.071	0.023	0.206
*Specific Patient cases.*	−0.092	−0.214	−0.104	0.061	−0.050	0.007	0.088
*Own responibility in the DCD process.*	−0.094	−0.162	−0.145	−0.159	−0.111	−0.257	−0.139
*Thoughts about DCD - Exciting*	0.025	0.021	−0.095	0.020	−0.030	0.118	−0.077
*Thoughts about DCD - unpleasant*	0.056	0.043	0.023	0.074	0.086	−0.058	0.074
*Thoughts about DCD - Informative*	0.094	0.124	0.101	−0.028	−0.086	−0.085	0.021
*Thoughts about DCD - unethical*	−0.021	−0.120	−0.158	0.041	−0.020	0.241	0.065
*Thoughts about DCD - interesting*	0.216	0.220	0.211	−0.118	−0.259	−,333^*^	−0.066
*Will the identification of potential DCD patients differ from potiential DBD patients?*	0.142	0.100	0.129	0.091	−0.066	−0.072	0.038
*Will the care of a DCD patient differ compared to the care of a DBD patient? *	−0.135	−0.181	−0.104	−0.081	−0.067	−0.051	−0.096
*Will the encounter of relatives to DCD patients differ compared to relatives of DBD patients? *	,296^*^	,317^*^	0.251	−0.102	0.028	−0.158	0.100
*Will the composition of the care team be different with DCD patients compared to DBD patients?*	0.203	0.246	,282^*^	0.137	0.188	0.035	,297^*^
*How important do you think it is to have physician involvement in the care of a DCD patient?*	0.096	0.176	0.133	−0.051	0.108	0.209	0.059

** Correlation is significant at the 0.01 level (2-tailed).

* Correlation is significant at the 0.05 level (2-tailed).

**Table:** 

Variable	To what extent have you followed the public debate on DCD?	Do you have unaswered questions refardning DCD?	Are you aware of the change in legislation in Sweden that enables the identification of DCD donors?	Is DCD something discussed in your department?	Ethical issues	Implementation issues
*Age*	−0.201	0.012	−0.211	−0.006	0.000	0.238
*Number of years as a RN*	−0.176	−0.045	−0.099	0.160	0.164	0.139
*No of years in ICU*	−0.216	−0.047	−0.121	0.170	0.094	0.094
*Experience of caring for an organ donator*	0.093	0.004	0.036	−0.156	−0.066	0.007
*Education from Organisation For Organ donation (OFO)*	,423^**^	−0.258	,283^*^	,280^*^	,386^*^	,373^*^
*Other organ donor education*	0.242	−0.216	0.133	,315^*^	−0.037	0.144
* What level of knowledge do you have of Donation after Circulatory Death (DCD)?*	,512^**^	−,425^**^	,338^*^	,298^*^	,366^*^	,389^*^
*To what extent have you followed the public debate on DCD?*	1.000	−,409^**^	,371^**^	,311^*^	,359^*^	0.318
*Do you have unaswered questions refardning DCD?*	−,409^**^	1.000	−,502^**^	−0.188	−,427^*^	−,429^*^
*Are you aware of the change in legislation in Sweden that enables the identification of DCD donors?*	,371^**^	−,502^**^	1.000	,344^*^	0.266	0.327
*Is DCD something discussed in your department?*	,311^*^	−0.188	,344^*^	1.000	0.276	,370^*^
*Ethical issues.*	,359^*^	−,427^*^	0.266	0.276	1.000	0.155
*Possibility to implement DCD.*	0.318	−,429^*^	0.327	,370^*^	0.155	1.000
*Practical issues regarding implementation.*	0.250	−,380^*^	,412^*^	0.169	,436^**^	0.144
*Personal opinion about DCD.*	0.264	−,471^**^	0.302	0.293	,579^**^	0.210
*Specific Patient cases.*	0.082	−0.213	−,364^*^	−0.298	0.066	−0.007
*Own responibility in the DCD process.*	−0.105	,354^*^	−,511^**^	−0.232	−0.278	−,424^*^
*Thoughts about DCD - Exciting*	0.045	−0.022	0.203	0.117	0.037	0.199
*Thoughts about DCD - unpleasant*	−,348^*^	−0.007	−0.065	0.030	−0.090	0.169
*Thoughts about DCD - Informative*	0.020	−0.017	0.166	0.016	0.217	0.210
*Thoughts about DCD - unethical*	0.136	−0.004	−0.036	0.156	−0.189	−0.007
*Thoughts about DCD - interesting*	−0.160	−0.034	−0.124	−0.265	0.093	−0.108
*Will the identification of potential DCD patients differ from potiential DBD patients?*	−0.020	0.228	−0.103	−0.052	−0.224	−0.310
*Will the care of a DCD patient differ compared to the care of a DBD patient? *	−,318^*^	0.156	−0.035	−0.178	−0.224	−0.145
*Will the encounter of relatives to DCD patients differ compared to relatives of DBD patients? *	−0.020	−0.100	0.068	0.020	,430^**^	0.108
*Will the composition of the care team be different with DCD patients compared to DBD patients?*	0.017	−0.098	0.222	0.084	0.244	0.165
*How important do you think it is to have physician involvement in the care of a DCD patient?*	0.068	0.088	0.129	,324^*^	0.189	0.253

** Correlation is significant at the 0.01 level (2-tailed).

* Correlation is significant at the 0.05 level (2-tailed).

**Table:** 

Variable	Practical issues regarding implementation	Personal opinion about DCD	Specific Patient Cases	Own responibility in the DCD process	Thoughts about DCD - Exciting	Thoughts about DCD - unpleasant
*Age*	−0.218	0.026	−0.092	−0.094	0.025	0.056
*Number of years as a RN*	−0.196	0.159	−0.214	−0.162	0.021	0.043
*No of years in ICU*	−0.201	0.127	−0.104	−0.145	−0.095	0.023
*Experience of caring for an organ donator*	0.239	0.201	0.061	−0.159	0.020	0.074
*Education from Organisation For Organ donation (OFO)*	0.210	0.071	−0.050	−0.111	−0.030	0.086
*Other organ donor education*	,428^*^	0.023	0.007	−0.257	0.118	−0.058
* What level of knowledge do you have of Donation after Circulatory Death (DCD)?*	,339^*^	0.206	0.088	−0.139	−0.077	0.074
*To what extent have you followed the public debate on DCD?*	0.250	0.264	0.082	−0.105	0.045	−,348^*^
*Do you have unaswered questions refardning DCD?*	−,380^*^	−,471^**^	−0.213	,354^*^	−0.022	−0.007
*Are you aware of the change in legislation in Sweden that enables the identification of DCD donors?*	,412^*^	0.302	−,364^*^	−,511^**^	0.203	−0.065
*Is DCD something discussed in your department?*	0.169	0.293	−0.298	−0.232	0.117	0.030
*Ethical issues.*	,436^**^	,579^**^	0.066	−0.278	0.037	−0.090
*Possibility to implement DCD.*	0.144	0.210	−0.007	−,424^*^	0.199	0.169
*Practical issues regarding implementation.*	1.000	,373^*^	0.007	−,393^*^	0.144	0.010
*Personal opinion about DCD.*	,373^*^	1.000	0.050	−0.306	−0.023	−0.110
*Specific Patient cases.*	0.007	0.050	1.000	0.159	−0.007	−0.088
*Own responibility in the DCD process.*	−,393^*^	−0.306	0.159	1.000	−0.152	−0.196
*Thoughts about DCD - Exciting*	0.144	−0.023	−0.007	−0.152	1.000	0.209
*Thoughts about DCD - unpleasant*	0.010	−0.110	−0.088	−0.196	0.209	1.000
*Thoughts about DCD - Informative*	0.023	0.167	0.050	−0.167	,312^*^	−0.072
*Thoughts about DCD - unethical*	0.007	0.050	−0.061	−0.134	0.183	0.240
*Thoughts about DCD - interesting*	−0.018	0.000	0.156	0.043	−0.078	−0.066
*Will the identification of potential DCD patients differ from potiential DBD patients?*	−,344^*^	−0.187	0.145	0.320	−0.066	−0.031
*Will the care of a DCD patient differ compared to the care of a DBD patient? *	−0.108	−0.258	0.117	−0.043	0.188	,540^**^
*Will the encounter of relatives to DCD patients differ compared to relatives of DBD patients? *	−0.108	,387^*^	0.117	−0.043	,381^**^	0.048
*Will the composition of the care team be different with DCD patients compared to DBD patients?*	0.204	0.201	−0.167	−0.222	0.188	0.147
*How important do you think it is to have physician involvement in the care of a DCD patient?*	0.239	0.201	−,470^**^	−0.159	0.276	0.091

** Correlation is significant at the 0.01 level (2-tailed).

* Correlation is significant at the 0.05 level (2-tailed).

**Table:** 

Variable	Thoughts about DCD - informative	Thoughts about DCD - unethical	Thoughts about DCD - Interesting	Will the identification of potential DCD patients differ from potiential DBD patients?	Will the care of a DCD patient differ compared to the care of a DBD patient?
*Age*	0.094	−0.021	0.216	0.142	−0.135
*Number of years as a RN*	0.124	−0.120	0.220	0.100	−0.181
*No of years in ICU*	0.101	−0.158	0.211	0.129	−0.104
*Experience of caring for an organ donator*	−0.028	0.041	−0.118	0.091	−0.081
*Education from Organisation For Organ donation (OFO)*	−0.086	−0.020	−0.259	−0.066	−0.067
*Other organ donor education*	−0.085	0.241	−,333^*^	−0.072	−0.051
* What level of knowledge do you have of Donation after Circulatory Death (DCD)?*	0.021	0.065	−0.066	0.038	−0.096
*To what extent have you followed the public debate on DCD?*	0.020	0.136	−0.160	−0.020	−,318^*^
*Do you have unaswered questions refardning DCD?*	−0.017	−0.004	−0.034	0.228	0.156
*Are you aware of the change in legislation in Sweden that enables the identification of DCD donors?*	0.166	−0.036	−0.124	−0.103	−0.035
*Is DCD something discussed in your department?*	0.016	0.156	−0.265	−0.052	−0.178
*Ethical issues.*	0.217	−0.189	0.093	−0.224	−0.224
*Possibility to implement DCD.*	0.210	−0.007	−0.108	−0.310	−0.145
*Practical issues regarding implementation.*	0.023	0.007	−0.018	−,344^*^	−0.108
*Personal opinion about DCD.*	0.167	0.050	0.000	−0.187	−0.258
*Specific Patient cases.*	0.050	−0.061	0.156	0.145	0.117
*Own responibility in the DCD process.*	−0.167	−0.134	0.043	0.320	−0.043
*Thoughts about DCD - Exciting*	,312^*^	0.183	−0.078	−0.066	0.188
*Thoughts about DCD - unpleasant*	−0.072	0.240	−0.066	−0.031	,540^**^
*Thoughts about DCD - Informative*	1.000	0.028	,328^*^	0.041	−0.077
*Thoughts about DCD - unethical*	0.028	1.000	−0.114	−0.091	0.081
*Thoughts about DCD - interesting*	,328^*^	−0.114	1.000	0.017	−,283^*^
*Will the identification of potential DCD patients differ from potiential DBD patients?*	0.041	−0.091	0.017	1.000	0.251
*Will the care of a DCD patient differ compared to the care of a DBD patient? *	−0.077	0.081	−,283^*^	0.251	1.000
*Will the encounter of relatives to DCD patients differ compared to relatives of DBD patients? *	0.263	0.102	0.057	,301^*^	0.247
*Will the composition of the care team be different with DCD patients compared to DBD patients?*	0.008	0.081	−0.089	0.251	0.089
*How important do you think it is to have physician involvement in the care of a DCD patient?*	0.049	0.051	−0.146	0.022	−0.011

** Correlation is significant at the 0.01 level (2-tailed).

* Correlation is significant at the 0.05 level (2-tailed).

**Table:** 

Variable	Will the encounter of relatives to DCD patients differ compared to relatives of DBD patients?	Will the composition of the care team be different with DCD patients compared to DBD patients?	How important do you think it is to have physician involvement in the care of a DCD patient?
*Age*	,296^*^	0.203	0.096
*Number of years as a RN*	,317^*^	0.246	0.176
*No of years in ICU*	0.251	,282^*^	0.133
*Experience of caring for an organ donator*	−0.102	0.137	−0.051
*Education from Organisation For Organ donation (OFO)*	0.028	0.188	0.108
*Other organ donor education*	−0.158	0.035	0.209
* What level of knowledge do you have of Donation after Circulatory Death (DCD)?*	0.100	,297^*^	0.059
*To what extent have you followed the public debate on DCD?*	−0.020	0.017	0.068
*Do you have unaswered questions refardning DCD?*	−0.100	−0.098	0.088
*Are you aware of the change in legislation in Sweden that enables the identification of DCD donors?*	0.068	0.222	0.129
*Is DCD something discussed in your department?*	0.020	0.084	,324^*^
*Ethical issues.*	,430^**^	0.244	0.189
*Possibility to implement DCD.*	0.108	0.165	0.253
*Practical issues regarding implementation.*	−0.108	0.204	0.239
*Personal opinion about DCD.*	,387^*^	0.201	0.201
*Specific Patient cases.*	0.117	−0.167	−,470^**^
*Own responibility in the DCD process.*	−0.043	−0.222	−0.159
*Thoughts about DCD - Exciting*	,381^**^	0.188	0.276
*Thoughts about DCD - unpleasant*	0.048	0.147	0.091
*Thoughts about DCD - Informative*	0.263	0.008	0.049
*Thoughts about DCD - unethical*	0.102	0.081	0.051
*Thoughts about DCD - interesting*	0.057	−0.089	−0.146
*Will the identification of potential DCD patients differ from potiential DBD patients?*	,301^*^	0.251	0.022
*Will the care of a DCD patient differ compared to the care of a DBD patient? *	0.247	0.089	−0.011
*Will the encounter of relatives to DCD patients differ compared to relatives of DBD patients? *	1.000	,342^*^	0.154
*Will the composition of the care team be different with DCD patients compared to DBD patients?*	,342^*^	1.000	0.169
*How important do you think it is to have physician involvement in the care of a DCD patient?*	0.154	0.169	1.000

** Correlation is significant at the 0.01 level (2-tailed).

* Correlation is significant at the 0.05 level (2-tailed).

Working experience from an ICU correlated significantly (*r* = .282, *p* = .045) with thoughts about differences between DBD and DCD from a care team perspective. Due to personal views, a significant correlation was seen between “Seeing DCD—as unpleasant” and differences in caring of DBD and DCD patients (*r* = .540, *p* = .00) and not following the public debate on DCD (*r* = −.348). However, no significant correlations were seen regarding thoughts about DCD as unethical.

Previous education and/or more responsibility concerning the donor process correlated significantly with a wish to discuss specific patient cases during DCD education (*r* = .697, *p* = .00). Practical questions about the implementation of DCD correlated significantly with ethical questions from the respondents (*r* = .436, *p* = .009). Additionally, personal opinions, ethics, and ethics in discussion forums correlated positively (*r* = .579, *p* = .00). Discussions of special patient cases and unit-related discussions were significantly correlated with having support from physicians (*r* = .324, *p* = .020 and *r* = −.470, *p* = .004, respectively).

There was also a significant correlation (*r* = .324, *p* = .020) between participants working in ICUs where the DCD process is discussed and thoughts about the importance of the commitment of the physicians in the care of the DCD patient/DCD process. In Table 6 all correlation analyses are presented.

#### Nurses’ Views on DCD

The participants could add free-text comments to 14 of the questions and 4 (four) questions had only open answers as an alternative. All free-text comments and the open-answer questions were qualitatively and narratively assessed and merged into four categories: *The importance of the team, Need for ethical discussions, Increased knowledge of DCD,* and *Unanswered questions and unmet needs.*

##### The Importance of the Team

The nurses described and emphasized donor care as teamwork where everyone is important. Without teamwork and/or commitment from the physicians, the DCD process would be unsafe or in the worst case, stopped.

“*Without proper medical involvement, it will not be good at all, or eventually no DCD at all*,” as one nurse described.

Close teamwork between local units, for example, the surgery unit and the ICU, was also highlighted by the nurses.

### Need for Ethical Discussions

When the ICU nurses reflected on DCD, interesting, exciting, and instructive were highlighted. There was also a wish for further ethical discussions. The respondent quotations below express these sentiments:

“*Not unethical, but there has to be room for ethical issues.*”

“*I am missing the ethical discussions about DCD. Especially those who work with this; they have not mentioned anything about the ethics, which I believe, is unfortunate.*”

Most important during the implementation phase, was to receive clear information about DCD and the DCD process. DCD was also an area of discussion at the reported units.

#### Increased Knowledge of DCD

To gain knowledge and information about DCD, local information sessions at the units and information from co-workers from the pilot DCD project and/or regional organ donor specialist nurses were highlighted. Furthermore, one unit had DCD as the “theme of the month.” The benefit of interprofessional education, exchange of knowledge from already existing DCD team and a wish for clear guidelines and time for ethical discussions were highlighted for contributing to the future implementation of DCD. Interprofessional simulations/scenario training, project groups and the same education for all professions at the unit were also highlighted. Ethical discussions were also seen as important. However, ethical approval seemed to be forgotten during education sessions on DCD. One nurse reflected:

“*Mostly, just the fact that you have to educate everyone in the workplace, not only those who work within the local donation groups*.”

#### Unanswered Questions and Unmet Needs

In the open answers, where DCD-specific questions were presented, care of the relatives, as well as practical questions like “*What kind of organs a DCD donor can donate*,” “*How is the patient reported dead?*,” “*cardiopulmonary-regulations*,” “*the time-aspect and inclusion criteria for DCD*” were mentioned. One participant described the situation as:

“*Difficult to pose a specific question before I have had a patient. The questions usually come in connection with a present case*.”

Other nurses narrated, that today's knowledge about DCD is weak:

“*We need to read exactly what it means if we will start with it*.”

### Integrative Analysis

The quantitative results show that most ICU nurses had limited knowledge of DCD, with over half reporting low or no understanding and highlighted significant gaps in knowledge and the open answers pointed out the need for information, clearer guidelines, and interprofessional education to address these gaps. Significant relationships emerged between background variables—such as age, experience, and education—and nurses’ knowledge and attitudes toward DCD. Nurses with more education had higher knowledge and more positive attitudes. The open answers illustrated how education and experience shaped nurses’ perceptions and ethical considerations. Overall, nurses highlighted the importance of team commitment, ongoing ethical discussions, and the need for more detailed knowledge, pointing to key areas for improvement in education and support systems regarding DCD. The complexity behind nurses’ attitudes also underscores the necessity for comprehensive education and support to effectively implement DCD practices.

## Discussion

The present study aimed to determine and describe Swedish ICU nurses’ knowledge, attitudes, and views on DCD before a potential implementation of the DCD process. Despite diverse knowledge about the DCD process, all the participating nurses were positive about the implementation of DCD in their units. However, according to the nurses, there are several key aspects, such as the importance of the team and ethical perspectives that need to be discussed to facilitate the national DCD implementation.

### Knowledge

The need for increased knowledge about DCD was highlighted in the results, with reflections about the new organ donation regulation in 2018, as well as other reflections about the DCD donor process. Furthermore, most of the participants in the present study (70.5%) reflected on the differences regarding the identification of a donor patient depending on the process, DBD or DCD. These findings are in line with studies highlighting difficulties in areas like non-perfusion time (Fleming & Thomson, 2018), and time-limit-related aspects of organ donation (Cooper, 2023).

Differences between the clinical aspects of DBD and DCD donations (Cooper, 2017; Kondori et al., 2021), therefore, need to be highlighted and clarified. However, the results of our study showed a correlation between ICU nurses’ working experience and thoughts about differences in the identification of a suitable donor patient. Therefore, continuous education and discussions about DCD and DBD need to be implemented in clinical settings where also ICU nurses’ experiences should be considered. According to Cooper (2023), it is evident to discuss the ethical perspectives of DCD, especially when the potential DCD donor patient does not qualify as a donor patient in the final stage. This needs to be emphasized during education, ethical discussions, and simulations. The results showed that previous education on organ donation positively correlated to an interest in ethical discussions. Nurses could encounter difficulties in their ethical conduct due to their difficult work environment. A structured implementation may lead to a safer work environment and will help to understand the barriers to the process (Davies et al., 2010). The nurses may have ethical doubts, and it is important to give extra time for open discussions before a new concept can be implemented (Goethals et al., 2010; Gripewall et al., 2022).

The results also showed that the level of knowledge about the new donation regulation (SFS, 2018, p. 307) was low among the participants. This stresses the importance of increased knowledge about the DCD process and conducting ethical discussions before end-of-life decisions. Furthermore, increased knowledge of DCD is necessary when searching in the Donor Registry before the patient is declared brain-dead (SFS, 2018, p. 307). This is pivotal for a possible DCD process (National Board of Health and Welfare, 2022). Additionally, the new regulation (SFS, 2022, p. 502), opened for organ conservatory treatment before the patient is declared dead optimizes the conditions for a good outcome regarding DCD donated organs (Foss et al., 2018; Klosiewicz et al., 2017; Puslecki et al., 2017). The new regulations make DCD implementation possible (Sweden National Board of Health and Welfare, 2022), and it is important to discuss, understand and reflect on this as a nurse and team member, and to acknowledge that the new concept of DCD can be perceived as difficult to understand (Manara et al., 2012). The implementation of DCD will lead to new ethical challenges that must be considered during implementation (Cooper, 2023). Zeiler et al. (2008) state that knowledge about the process and continued information needs to be given to relatives and the donor care team may contribute to a supporting and caring culture (Nyholm et al., 2018). Cooper (2017, 2023) notes that DCD is highly ethically problematic. Furthermore, the importance of enhanced knowledge among staff in ICU regarding organ donation cannot be underestimated to increase the number of donated organs (Alshammari & Brown, 2023). An open discussion in the care team about death as well as information for relatives will also help to increase the understanding of the DCD process and the ethical aspects*.*

### Attitudes and Views on DCD

The importance of the team was emphasized, and teamwork at the bedside was highlighted in the results. Multiple barriers, such as disinterest from physicians or dysfunctional cooperation between different units were seen in the results, which are confirmed in the previous literature (Gripewall et al., 2022; Kondori et al., 2021; Milross et al., 2022). Healthcare professionals highlight a need for team-based simulations. Concerning the DCD process, a correlation was seen between the importance of an attending physician and nurses’ engagement in the DCD process (Foss et al., 2018), The comments in the open answers confirm the need for team simulations and physician commitment. Rosén et al. (2018) found that suboptimal teamwork processes are a public health issue. The present study shows that the understanding of the DCD process was rather low, as well as interest in the debate in society.

### Associations Between Nurses’ Demographic Characteristics, Knowledge, and Attitudes

No significant correlations were seen regarding thoughts about DCD as an unethical process. Although, ethical discussions about DCD are well-known worldwide and reflect the importance of understanding the DCD process in both medical and ethical ways (Cooper, 2017, 2023; Guzik-Makaruk et al., 2019; Sade, 2011; Tantillo et al., 2019; Zeiler et al., 2008). In the results, uncertainty about the identification process of the organ donor patient emerged, as well as reflections about what kind of organs could be donated. Nurses with higher knowledge and education in organ donation were interested in ethics, following the public debate on DCD and were more positive toward the implementation of DCD compared to nurses without donor education. These findings are in line with previous research which states that the organ donation process and the DCD process will be a non-everyday tasks for the ICU nurse (Cooper, 2023; Gripewall et al., 2022; Milross et al., 2022; Moghadam et al., 2018). This correlation underpins the importance of a wider national organ donor training and education program among nurses working in ICUs.

### Strengths and Limitations

We chose to employ a mixed-methods design in this study to obtain a comprehensive understanding of Swedish ICU nurses’ knowledge, attitudes, and views on DCD before a national implementation. The cross-sectional design allows for the collection and analysis of numerical data to have insights into nurses’ knowledge levels and attitudes (Polit & Beck, 2022). The design cannot capture the depth of nurses’ experiences and their views and ethical views on DCD why also open-ended questions were used in the questionnaire. By combining these approaches, the study to not only quantifies knowledge and attitudes but also understands the underlying reasons and context behind (Creswell & Plano Clark, 2018). A strength of this study is that data was retrieved in ICUs in Sweden before the national implementation of DCD. This means that the collected data is not influenced by education and/or information campaigns. The data was also collected before the COVID-19 pandemic, and the result is therefore not affected by the post-pandemic effects on the Swedish healthcare system. Furthermore, to our knowledge, it is the first study on nurses’ views on DCD in a Swedish context.

The sample size is a limitation of this study, as well as the limited number of participants that responded. The response rate was low (35%) which could be due to the short response time of two (2) weeks and only one reminder. In clinical surveys, a three-week response time can be considered as short, as many nurses work irregularly, and few might have time to answer. However, the answers were recruited from frontline intensive care nurses, which is why the results of this study can be considered valid even if the response rate was low. Another large limitation was that we had to develop a study-specific questionnaire. To our knowledge, no validated tool in the specific research area could be found at the time of data collection. Furthermore, our study-specific tool contained open and closed questions and only three questions were of Likert's scale type which is a weakness as the robustness of the tool only can be assessed by pre-test and face validity. The pre-test and face validity showed agreement and only minor changes of language character was made to the tool. However, the open answers, and the possibility to comment even in fixed-answer questions, allowed the participants to further develop their answers. As there was no existing questionnaire in this specific uncharted area, the questionnaire was based on research from the field and based from the Swedish National Council for Organs, Tissues, Cells and Blood. The questionnaire was then pre-tested, to increase its validity (Polit & Beck, 2022) and the questions of Likert's scale showed an acceptable internal consistency considering they were few.

The study's trustworthiness can be considered agreeable as the data from the free-text answers was derived from non-dependable participants and the settings consisted of ICUs in larger hospitals in Sweden where organ donors are cared for. Data was collected from multiple settings of different ICUs to ensure the study's credibility and the transferability of the findings. The thorough stepwise analysis made by the research group accounted for the study's confirmability (Lincoln & Guba, 1985). Authenticity (Guba & Lincoln, 1985) was also considered as the data collection was carried out in ICUs where organ donor patients are usually cared for. The clarity and transparency of reporting how the quantitative and qualitative components are integrated was described, as well as the integration process of how the results complemented each other. This is important to ensure the quality assessments (O’Cathain et al., 2008).

### Implications for Practice

The results of this study highlight a need for an interprofessional, national implementation plan. Support from the organization when implementing DCD is also evidently needed. In addition, team-based education and simulation is highly recommended. Additionally, ethical discussions and aspects of DCD *and* caring are pivotal at team- and management levels, to understand the complexity of the DCD process and promote the confidence and willingness to identify potential DCD patients at ICUs in Sweden.

## Conclusion

The present study determined and described Swedish ICU nurses’ knowledge and attitudes toward DCD before a national implementation of the DCD process. More than 50% had limited knowledge about DCD, and those with previous education in organ donor care had higher knowledge and displayed a more positive attitude toward the implementation of DCD in their units. Furthermore, they followed the public debate and discussed different aspects of the implementation process, whereas nurses without specific donor education were more reluctant to the implementation. The importance of interprofessional teamwork and discussions from ethical perspectives were key aspects that were emphasized by the nurses to be discussed to facilitate the national DCD implementation. The integrated analysis pointed out the importance of targeted education, clear guidelines, and ethical discussions in preparing ICU nurses for the implementation of DCD in Sweden. These findings may lead to a higher motivation to identify potential DCD donor patients and to implement the DCD process, which is needed due to a global lack of transplantable organs.
